# Antegrade delivery of a thoracic endovascular stent graft via axillary access with retrograde in situ laser fenestrations

**DOI:** 10.1016/j.jvscit.2026.102199

**Published:** 2026-02-26

**Authors:** Mitri K. Khoury, Alvaro Mendez, Shiv Patel, Hasan Aldailami

**Affiliations:** a Division of Vascular and Endovascular Surgery, Arizona State University, Tempe; b Division of Vascular and Endovascular Surgery, HonorHealth Heart, Scottsdale

**Keywords:** Aorta, Aneurysm, Endovascular, Laser, Fenestration

## Abstract

Thoracic endovascular aortic repair is typically done through a retrograde transfemoral approach. However, it is not uncommon for the femoral or iliac arteries to be heavily diseased, making retrograde delivery difficult. This has traditionally been addressed through open and endovascular conduits. We present a case of a patient with a ligated infrarenal aorta and a pseudoaneurysm arising from a supraceliac to bi-iliac bypass. This was subsequently treated via antegrade delivery of a thoracic endovascular aortic repair device via axillary access with in situ laser fenestrations for the iliac limbs.

Thoracic endovascular aortic repair (TEVAR) has now become the treatment of choice for aortic pathology arising from the descending thoracic aorta.^1^ Delivery of a TEVAR device is typically performed via a transfemoral approach, either open or percutaneously. There are times when retrograde delivery of a TEVAR via a transfemoral approach is not possible owing to the poor caliber of the iliofemoral system, severe iliac tortuosity, or extensive atherosclerosis.^2^ In these situations, open or endovascular conduits are used to assist with delivery of the device into the descending thoracic aorta.^3^ Transaxillary delivery transcatheter aortic valve replacement has been well-described for patients with severe iliofemoral disease.^4^ However, this access has only been described in select cases for treatment of the thoracic and abdominal aorta.^5^, ^6^, ^7^, ^8^ We present the case of a patient with a ligated infrarenal aorta and a pseudoaneurysm arising from a supraceliac to bi-iliac bypass. This was subsequently treated by delivering a TEVAR device from an axillary approach with in situ laser fenestrations for the iliac limbs. The patient provided written informed consent for the report of his case details and imaging studies.

## Case report

A 73-year-old man with a history of hyperlipidemia, hypertension, colitis, small bowel obstructions, and hepatitis C presented with pseudoaneurysms arising from the proximal and distal anastomoses of a prior supra celiac to bi-iliac bypass. The patient had a complicated vascular history in which his infrarenal aorta was ligated and a right axillary-to-bifemoral bypass was performed after being stabbed multiple times in the abdomen approximately 40 years ago. His axillary-to-bifemoral bypass was eventually revised to a supraceliac-to-bilateral common iliac bypass shortly after his axillary-to-bifemoral bypass. Approximately 2 years before his current presentation, he presented with low back and hip pain and was found to have an 8-cm pseudoaneurysm arising from the right common iliac artery where his prior anastomosis was performed. A surgeon at that time had placed a 28 × 140 mm Ovation iX limb endoluminal graft (Endologix) from the prior graft into the right common iliac artery.

**Fig 1:**
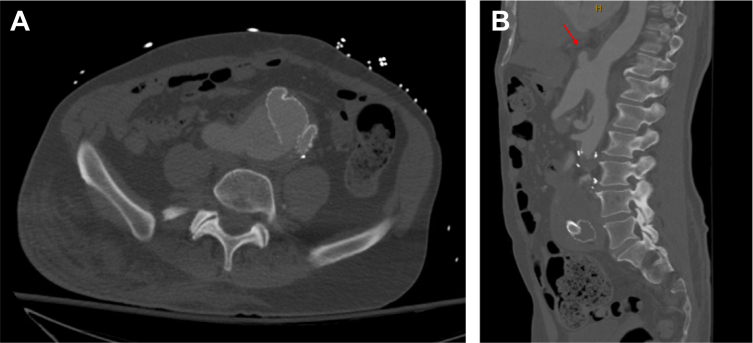
Computed tomography images demonstrating **(A)** a pseudoaneurysm arising from the right iliac limb in the axial plane and **(B)** a pseudoaneurysm arising from the supraceliac anastomosis (*red arrow*) in the coronal plane.

He now presented to our service with an 11-cm pseudoaneurysm arising from the prior anastomosis with the previously placed iliac limb completely dislodged from the right common iliac artery (Fig 1, *A*). He also had a pseudoaneurysm arising from the supraceliac anastomosis that measured 2.2 × 3.3 cm, which had grown from the year prior (Fig 1, *B*). A tagged white blood cell (WBC) scan was obtained and demonstrated uptake in the pseudoaneurysm in the right iliac pseudoaneurysm but no uptake in the pseudoaneurysm arising from the supraceliac anastomosis. Blood and urine cultures were negative. The C-reactive protein level was normal at 0.3 mg/dL. The patient was never febrile, nor did he ever have a leukocytosis. Both open and endovascular options were entertained to address the right iliac pseudoaneurysm. The endovascular option was felt to be better suited for this patient given his hostile abdomen.

**Fig 2:**
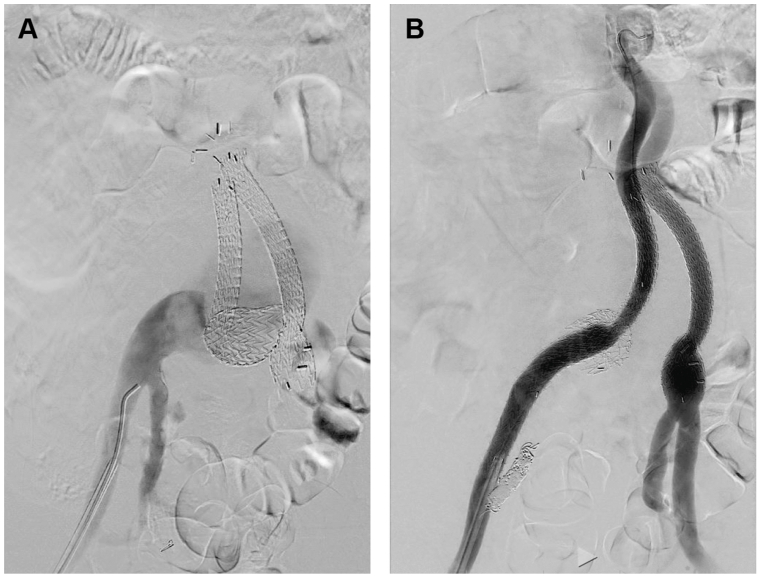
Intraoperative fluoroscopy demonstrating **(A)** a pseudoaneurysm arising from the right common iliac artery with the previously placed iliac limb dislodged and **(B)** a completion angiogram with resolution of the pseudoaneurysm via coiling of the internal iliac artery and limb extension into the external iliac artery.

The patient was taken to the operating room and the right common femoral artery was accessed percutaneously. The right internal iliac artery was subsequently coil embolized utilizing detachable coils (Penumbra). The right iliac limb was extended into the external iliac artery using two Gore 16 × 140 mm Iliac Limbs (W. L. Gore & Associates). A final angiogram demonstrated exclusion of the right pseudoaneurysm (Fig 2). The patient was treated with empiric vancomycin and piperacillin-tazobactam until all his cultures resulted negative. Given that an infection of the pseudoaneurysm could not be ruled in or out, he was treated with oral sulfamethoxazole/trimethoprim for 3 months. After a 3-month course of sulfamethoxazole/trimethoprim, the patient had a repeat tagged WBC scan, which demonstrated resolution of the prior uptake. He continued to be afebrile with no leukocytosis. At this point, we prioritized treatment of his pseudoaneurysm arising from his supraceliac anastomosis.

**Fig 3:**
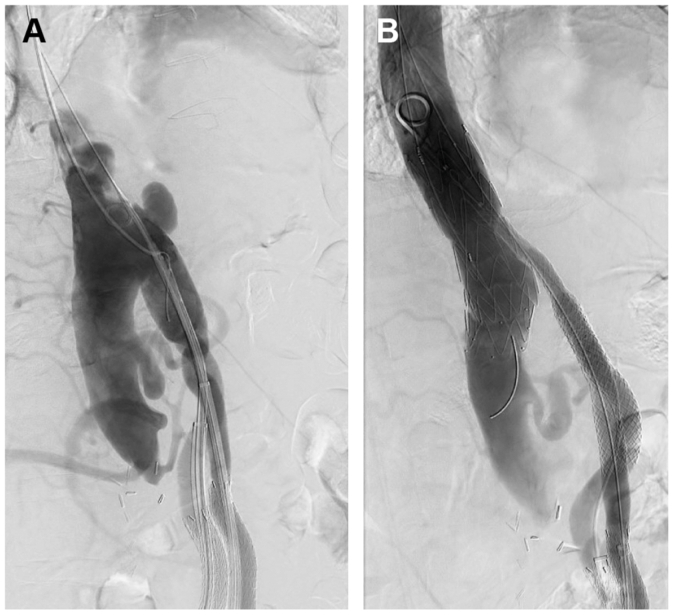
Intraoperative fluoroscopy demonstrating **(A)** a pseudoaneurysm arising from the supraceliac anastomosis and **(B)** completion angiography with resolution of the pseudoaneurysm following thoracic endovascular stent graft placement with laser in situ fenestrations for the iliac limbs.

**Fig 4:**
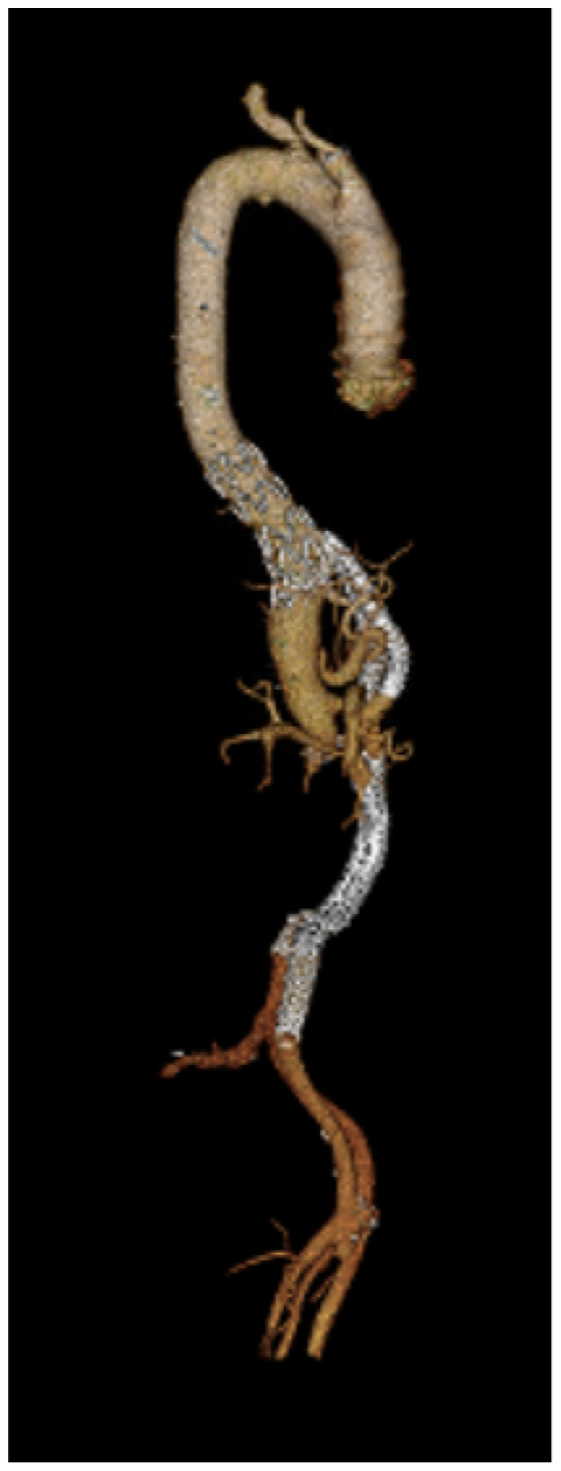
Three-dimensional reconstruction of the final repair on the 1-month postoperative scan.

The patient was brought to the operating room with the bilateral groins and chest prepped under general anesthesia. The bilateral groins were accessed percutaneously under ultrasound guidance and Gore DrySeal 12F × 33 cm sheaths (W. L. Gore & Associates) were placed into the common femoral arteries. The left axillary artery was controlled via open exposure through an infraclavicular incision. Given the patient had an infrarenal stump, the Lunderquist wire (Cook Medical) would be resting on this stump and could perforate it should too much force be applied during device delivery. Therefore, it was felt that delivering the device through a DrySeal would allow for a smoother delivery of the device. A Gore DrySeal 22F × 33 cm (W. L. Gore & Associates) was subsequently placed into the descending thoracic aorta. Two 8.5F Agilix Steerable sheaths (Abbot Vascular) were then placed into the main body of the bifurcated bypass graft from the femoral access. A 34 × 100 RelayPro (Terumo Aortic) was then deployed from the descending thoracic aorta into the supraceliac abdominal aorta. Next, laser fenestrations were performed for the bilateral iliac limbs through the steerable sheaths using the 2.3-mm TurboElite (Phillips). Two 7 × 79 VBXs (W. L. Gore & Associates) were then inflated within the fenestrations and were extended into each iliac limb using an 11 × 79 VBX (W. L. Gore & Associates). The distal aspect of each 11 × 79 VBX (W. L. Gore & Associates) were postdilated to 12 mm to match the diameter of the prior bypass. Completion angiogram demonstrated resolution of the pseudoaneurysm (Fig 3). The patient was discharged home on postoperative day 2. A computed tomography scan performed 1 month later demonstrated resolution of the pseudoaneurysm with a patent TEVAR and iliac limbs with no endoleak (Fig 4).

## Discussion

This case demonstrates the feasibility of performing a TEVAR through an antegrade transaxillary approach in a patient with unusual access. TEVAR is typically performed via a retrograde approach through the common femoral artery. However, there are a variety of reasons why retrograde delivery of a TEVAR may not be suitable, such as extensive atherosclerosis, vessel diameter, and severe tortuosity. Although both open and endovascular conduits were used to overcome the limitations of poor access, alternative access may be a better option in certain patients.

**Fig 5:**
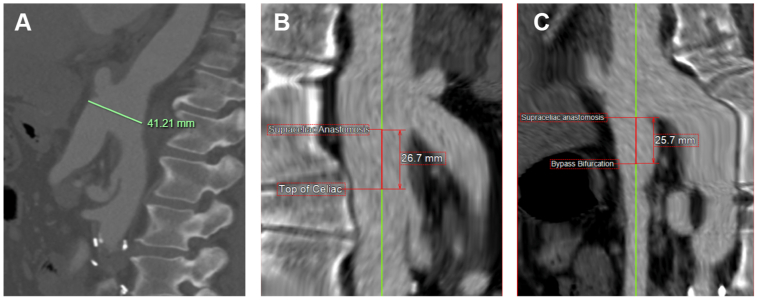
Measurements demonstrating **(A)** the distance between the supraceliac anastomosis and the proximal portion of the celiac artery, **(B)** the distance between the supraceliac anastomosis and bifurcation of the bypass, and **(C)** the distance between the posterior wall of the aorta and anterior aspect of the main body of the bypass.

Open reconstruction was considered but would have been morbid and physiologically demanding on the patient. Thus, various endovascular options were entertained, which included (1) retrograde delivery via femoral access of a TEVAR with a large fenestration for the visceral segment of the aorta, (2) branched endovascular aortic repair with branches oriented down the visceral segment of the aorta and cannulation of the branches via upper extremity access, and (3) transaxillary delivery of a TEVAR device with fenestrations for the iliac limbs. The concern with option 1 is that there would be a large endoleak arising from around the graft into the pseudoaneurysm because the distance between the posterior wall of the aorta to the anterior wall of the main body of the graft is approximately 41 mm (Fig 5, *A*). The concern with option 2 is that the branches would have been very long because the supraceliac to bi-iliac anastomosis was approximately 2.7 cm above the celiac (Fig 5, *B*). In addition, the main body of the bypass was only 2.5 cm in length, limiting distal seal (Fig 5, *C*). This would require a bifurcate piece into the iliac limbs, which would also increase the length of the branches. The axillary artery was free of atherosclerosis and measured to be approximately 10 mm in diameter. Further, the left vertebral artery arose directly off the aorta mitigating the risk of a stroke via this approach. Therefore, we elected to deliver a TEVAR through a left transaxillary approach with laser fenestrations for the iliac limbs as this would likely provide a durable repair and minimize complexity in an already unusual case. Transaxillary delivery may be an enticing option for patients with unsuitable access for retrograde delivery. The axillary artery is usually large in caliber, devoid of atherosclerosis, and not tortuous. However, the greatest concern with upper extremity access is the risk of stroke owing to emboli as one crosses the arch, which can be mitigated by utilizing left vs right upper extremity access.^9^ In our case, the left vertebral artery arose directly off the aorta, making stroke even less likely with left upper extremity access.

Device selection is an important consideration when performing an antegrade TEVAR. All TEVAR devices are designed to withstand downward forces from the heart via a retrograde delivery. Therefore, when performing an antegrade TEVAR, the device is upside down and the type of device fixation needs to be considered. Thus, we decided to choose a device that does not rely on active fixation but rather passive fixation via radial forces on to the aorta. Roselli et al^10^ have previously published outcomes regarding antegrade TEVAR. They described three different methods of delivery: transaxillary, ascending aortic delivery, and direct aortic replacement, with transaxillary being the least commonly used method. The most used devices were Zenith TX2 (Cook Medical) and Gore TAG (W. L. Gore & Associates), which rely on passive fixation. Among the 32 patients who had imaging follow-up, there was one type Ia endoleak within 6 months. However, it is unknown whether this was a result of device migration or not. Nonetheless, it is the authors' opinion that when performing an antegrade TEVAR, a device that primarily depends on passive fixation should be preferred.

We also used in situ laser fenestrations instead of performing physician modifications on the graft. First, this simplified the approach owing to the lack of need to orient the device in the paravisceral aorta with very limited working space given the ligated infrarenal aorta. In addition, the authors decided that retrograde in situ laser fenestration for the iliac limbs is similar in concept to retrograde in situ laser fenestration of the subclavian artery, which has been shown to have excellent patency.^11^ Therefore, it was decided that retrograde in situ laser fenestration would provide a durable outcome and simplify the repair in a patient with complicated anatomy.

This case also highlights the possibility of treating infected pseudoaneurysms via endovascular therapy and antibiotics. Prior studies have demonstrated the feasibility of this approach in selected patients.^12^, ^13^, ^14^ The authors thought a minimalistic approach to this patient would be beneficial, given there was no clinical signs or symptoms of infections (negative cultures, afebrile, and normal white blood cell count) other than a positive tagged WBC scan. If there was an infection, it was likely that the infection was indolent and could be treated with antibiotics and not wide debridement and reconstruction.

## Conclusions

Antegrade transaxillary delivery of a TEVAR is a feasible option for patients with unsuitable access for traditional retrograde delivery. Devices using passive fixation are a viable option.

## Funding

None.

## Disclosures

None.
